# Effects of Vegetarian Diets on Blood Pressure Lowering: A Systematic Review with Meta-Analysis and Trial Sequential Analysis

**DOI:** 10.3390/nu12061604

**Published:** 2020-05-29

**Authors:** Kai Wei Lee, Hong Chuan Loh, Siew Mooi Ching, Navin Kumar Devaraj, Fan Kee Hoo

**Affiliations:** 1 Department of Family Medicine, Faculty of Medicine and Health Sciences, Universiti Putra Malaysia, Serdang 43400, Selangor, Malaysia; lee_kai_wei@yahoo.com (K.W.L.); sm_ching@upm.edu.my (S.M.C.); knavin@upm.edu.my (N.K.D.); 2 Clinical Research Centre, Hospital Seberang Jaya, Ministry of Health Malaysia, Perai 13700, Penang, Malaysia; lohhongchuan@gmail.com; 3 Malaysian Research Institute on Ageing, University Putra Malaysia, Serdang 43400, Selangor, Malaysia; 4 Department of Medical Sciences, School of Healthcare and Medical Sciences, Sunway University, Bandar Sunway 47500, Selangor, Malaysia; 5 Department of Medicine, Faculty of Medicine and Health Sciences, University Putra Malaysia, Serdang 43400, Selangor, Malaysia

**Keywords:** hypertension, diet, vegan, vegetarian, plant-based diets

## Abstract

The beneficial effects of a vegetarian diet on blood pressure (BP) control have been reported in previous systematic reviews; however, so far, their relative effectiveness is not well established. Here, we performed a systematic review together with trial sequential analysis to determine the effect of a vegetarian diet on the reduction of blood pressure. We searched the randomized controlled trial (RCT) through Medline, PubMed and Cochrane Central Register. Fifteen eligible RCTs with 856 subjects were entered into the analysis. The pooled results demonstrated that vegetarian diet consumption significantly lowered the systolic blood pressure (weighted mean difference (WMD), −2.66 mmHg (95% confidence interval (CI) = −3.76, −1.55, *p* < 0.001) and diastolic BP was WMD, −1.69 95% CI = −2.97, −0.41, *p* < 0.001) as compared to an omnivorous diet. In subgroup analysis, a vegan diet demonstrated a greater reduction in systolic BP (WMD, −3.12 mmHg; 95% CI = −4.54, −1.70, *p* < 0.001) as compared with a lacto-ovo-vegetarian diet (WMD, −1.75 mmHg, 95% CI −5.38, 1.88, *p* = 0.05). The vegan diet has showed a similar trend in terms of diastolic blood pressure reduction (WMD, −1.92 mmHg (95% CI = −3.18, −0.66, *p* < 0.001) but those with a lacto-ovo-vegetarian diet showed no changes in diastolic BP reduction (WMD, 0.00, 95% CI = 0.00, 0.00), *p* = 0.432). In conclusion, vegetarian diets are associated with significant reductions in BP compared with omnivorous diets, suggesting that they may play a key role in the primary prevention and overall management of hypertension.

## 1. Introduction

It is estimated that about 26% of the world’s population (957–987 million) were affected with hypertension in 2000, with 333 million from developed countries and 639 million from developing countries [1]. It is an escalating trend, rising from 594 million in 1975 to 1.13 billion in 2015, and this mainly now occurs in low-income and middle-income countries. This global shifting has resulted from the increase in population growth and unprecedented process of aging [2].

High blood pressure or hypertension is defined as systolic blood pressure of 140 mmHg or higher, and diastolic blood pressure of 90 mmHg or higher [3]. It is an independent and major risk factor for cardiovascular disease and chronic kidney failure [4,5,6,7]. Patients with hypertension have a twofold to threefold higher risk for heart disease or stroke, as compared with those without hypertension [4,5]. In addition, patients with hypertension are more likely to develop type 2 diabetes, and patients with type 2 diabetes also have hypertension as well [8,9]. Patients having both hypertension and diabetes possess the highest risk of developing a stroke event and, in particular, having a fatal stroke [6]. On top of that, hypertension alone contributed about 29% of the underlying cause of end-stage renal disease [7].

Vegetarian diets are defined as dietary patterns that are determined by the level of animal food intake, and can be classified into several subtypes. Firstly, the vegan diet is defined as plant-based diet only; secondly, the lacto-ovo-vegetarian diet refers to diet that is without meat but may include eggs and/or dairy products; thirdly, the pescovegetarian diet may contain fish, but with other meats only taken < once/month; and the semivegetarian diet contains meats besides fish occasionally, but these are only taken < once/week [10]. Vegetarian diets generally have a higher diet quality than nonvegetarian diets [11]. This is due to the fact that the vegetarian diets have a higher portion of glutamic acid and plant-based protein, which has blood-pressure-lowering effect [12,13,14]. Besides, the higher fiber, antioxidants content, potassium content and lower saturated fat and sodium content in vegetarian diets can contribute to a lower body mass index and blood pressure readings [15,16,17]. Vegetarian diet reduces high blood pressure via several mechanisms, such as by improving blood viscosity, vasodilation and insulin sensitivity; by altering the baroreceptors, renin-angiotensin and sympathetic nervous system; by its anti-oxidant and anti-inflammatory properties and by changing the colony and strain of gut microflora [18,19,20,21,22,23]. Thus, many studies have shown that dietary patterns with a lower meat intake like the vegetarian diet is associated with a lower rate of non-communicable disease particularly hypertension [16,17] and contributed to a better health outcome and longer life expectancy.

However, to date, previous studies have offered conflicting results regarding this topic [24,25], and, to our knowledge, there has not been any quantitative attempt to summarize the precise effect of vegetarian diets on blood pressure reduction. Thus, we have performed a trial sequential analysis (TSA) to determine whether the currently available evidence was sufficient and conclusive on this important subject matter. A meta-analysis of studies with TSA methodology could elucidate the associations between vegetarian diets and blood pressure lowering from estimation of the effect size regarding the benefits of vegetarian diets consumption compared with omnivorous diets by looking at whether adequate sample power size of the randomized controlled trials have been achieved and whether the conclusions are valid or not.

## 2. Methods

The protocol was registered in the Medical Research and Ethics Committee, Ministry of Health Malaysia (registration number: NMRR-20-250-53210) on 25 February 2020. The reporting followed the Preferred Reporting Items for Systematic Reviews and Meta-analyses (PRISMA) guidelines [26].

### 2.1. Data Sources

We searched three major citation databases (Medline, PubMed and Cochrane Central Register of controlled trials) and included all relevant citations regardless of data of publication from inception to 6 January 2020. We also used forward and backward citation from the included studies to search for relevant studies. The search strategy is shown in Table S1.

### 2.2. Intervention and Control Group Definitions

The intervention group was defined as participants who were on a lacto-ovo-vegetarian diet (defined as those excluding meat, poultry and fish, and consuming eggs and dairy products) or a vegan diet (defined as excluding animal-derived food products such as meat, fish, poultry, eggs, and milk) for at least two weeks. The control group was defined as participants who were on an omnivorous diet (defined as those who consume meat, poultry, fish, eggs, and dairy products).

### 2.3. Study Selection

We included randomized controlled trials of two weeks’ follow-up duration comparing the effect of vegetarian diets (which included vegan to lacto-ovo-vegetarian) with non-vegetarian diets on systolic and diastolic blood pressure as a primary or secondary outcomes. The included studies had to be published in English or have an English translation. Studies were excluded if there was no vegetarian intervention, no nonvegetarian control, or no suitable outcome data.

### 2.4. Data Extraction

Two investigators (KWL and HCL) independently reviewed and extracted relevant data from each of the included studies. A standardized form was used to extract data on the following variables: first author, publication year, country, baseline characteristics of the study population, baseline and follow-up blood pressure levels, and changes of blood pressure in mean ± standard deviation. All discrepancies and disagreements were resolved through consensus.

### 2.5. Data Syntheses

Mean differences in systolic and diastolic blood pressure between patients assigned to receive the vegetarian and comparators diets were calculated using a meta-analysis with random-effects model and trial sequential analysis. Estimates of net changes in blood pressure associated with the consumption of vegetarian diets were combined using a random effects model and the result was reported with a 95% confidence interval with a two-sided *p* value of <0.05, which was considered as statistically significant. Meta-analyses were conducted using Open Meta Analyst software [27]. We performed trial sequential analysis for primary outcome with the random-effect (DL) model using the TSA software package [28]. Meta-analysis is the statistical procedure for combining data from multiple studies in order to determine overall trends, however meta-analysis may have type I errors due to systematic bias or random errors (for example, sparse data and repeated significance testing) [28]. TSA is a methodology that combines sample sizes of all included trials with the threshold of statistical significance, which quantifies the statistical reliability of data in the cumulative meta-analysis adjusting the significance levels for sparse data and repetitive testing on accumulating data [29]. Therefore, the results in meta-analysis are more reliable and conclusive with a TSA, as it helps to reveal insufficient information size and potential false positive results in the meta-analysis [30].

We used mean ± standard deviation (SD) to express our outcomes. If the mean difference and SD were not provided, the mean was calculated by subtracting the mean of baseline measurement from the corresponding mean of post intervention measurement; SD was imputed from the end point measurement. If the mean difference was provided, but the SD was not, the latter was imputed either from the end point measurement or calculated using the confidence intervals with the formulas “SQRT (sample size) × (upper confidence interval-lower confidence interval)/(T.INV.2T (0.05, $D$2-1) × 2)” proposed by [31]; this function was used to calculate estimated SD with Excel.

### 2.6. Risk of Bias Assessment

Included trials were independently assessed by two investigators (KWL and HCL) for risk of bias using the Cochrane Risk of Bias Tool [32,33]. Assessment was done across five domains of bias (Bias arising from the randomization process, bias due to deviations from intended interventions, bias due to missing outcome data, bias in measurement of the outcome and bias in selection of the reported result). The risk of bias was assessed as either low risk (proper methods taken to reduce bias), some concern (insufficient information provided to determine the bias level), or high risk (improper methods creating bias). All discrepancies and disagreement were resolved through consensus, or, where necessary, by the third (SMC) and fourth author (NKD). A graph and a summary of risk of bias were generated using Review Manager (Rev-Man), version 5.3 (Nordic Cochrane Conte, Cochrane Collaboration, Copenhagen, Denmark).

### 2.7. Grading of the Evidence

The Grading of Recommendations Assessment, Development, and Evaluation (GRADE) approach was used to assess the certainty of the evidence [34,35,36,37,38]. The certainty of the evidence was graded as high, moderate, low, or very low. Randomized controlled trials received an initial grade of high of evidence by default, or high inconsistency (I^2^ > 75%, *p* value < 0.05) [39], indirectness (presence of factors that limit the generalizability of the results) [40], imprecision (the 95% CI for weighted mean difference were wide) [41], and/or publication bias (significant evidence of small-study effects) [42].

## 3. Results

### 3.1. Search Results

**Figure 1:**
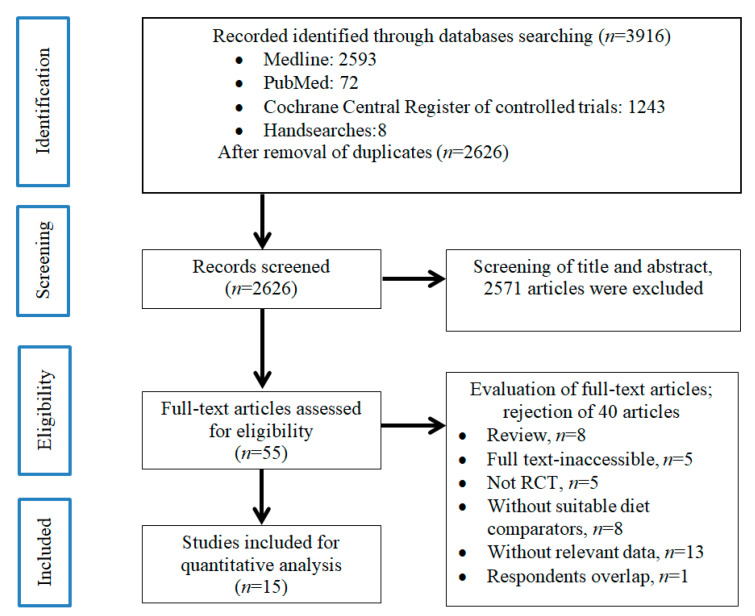
Preferred Reporting Items for Systematic Reviews and Meta-analyses (PRISMA) flow diagram of the literature screening process.

The literature search and selection process is shown in Figure 1. We identified a total of 2626 studies after removing duplicates, 2571 of which were excluded based on review of the title and/or abstract. The remaining 55 studies were retrieved and reviewed in full, of which 40 were excluded based on selection criteria. A total of 15 studies involving 856 individuals met the eligibility criteria and were included in the final analyses [43,44,45,46,47,48,49,50,51,52,53,54,55,56,57].

### 3.2. Trials Characteristics

Characteristics of the 15 included studies are shown in Table 1. Fourteen of the trials were conducted among adults [43,44,45,46,47,48,50,51,52,53,54,55,56,57] and one was in children [49]. The intervention group in five of the studies was receiving a lacto-ovo-vegetarian diet [47,52,54,55,56], and the intervention group in 10 of the studies was receiving a vegan diet [43,44,45,46,48,49,50,51,53,57]. Eight studies were conducted among participants with diabetes [43,44,46,48,50,51,53,57] and seven studies were among participants without diabetes [45,47,49,52,54,55,56]. More than half of studies were conducted in either the United States [43,44,45,46,47,49,50,51,53,56], Australia [52,54,55], New Zealand [57] or South Korea [48]. The clinical trials had a follow-up duration that ranged from three to 74 weeks. The overall baseline BP was 125.5 mmHg (108–149.5 mmHg)/75.3 mmHg (65–86 mmHg). The overall postinterventional BP was 122.6 mmHg (106.4–147 mmHg)/73.8 mmHg (64–83.5 mmHg).

### 3.3. Effect of Vegetarian Diets on Systolic Blood Pressure Lowering and Its Subgroup Analysis

The effect of vegetarian diets on lowering systolic blood pressure and subgroup analysis by diet subgroup, diabetes status, and country are shown in Table 2. A significant reduction in systolic blood pressure was observed in those taking vegetarian diets as compared to those on a control diet (weighted mean difference (WMD = −2.655, 95% CI = −3.758, −1.553)) among all participants, regardless of age groups. Similar results were also obtained among adult participants (WMD = −2.509, 95% CI = −3.630, −1.388). In subgroup analysis, we observed that a vegan diet (WMD = −3.118, 95% CI = −4.540, −1.696) had a greater reduction in systolic blood pressure compared to a lacto-ovo-vegetarian diet (WMD = −1.752, 95% CI = −5.382, 1.878). Similarly, there was a greater reduction in systolic blood pressure among participants without diabetes (WMD = −4.083, 95% CI = −7.684, −0.482) compared to those with diabetes (WMD = −1.625, 95% CI = −3.106, −0.144).

**Figure 2:**
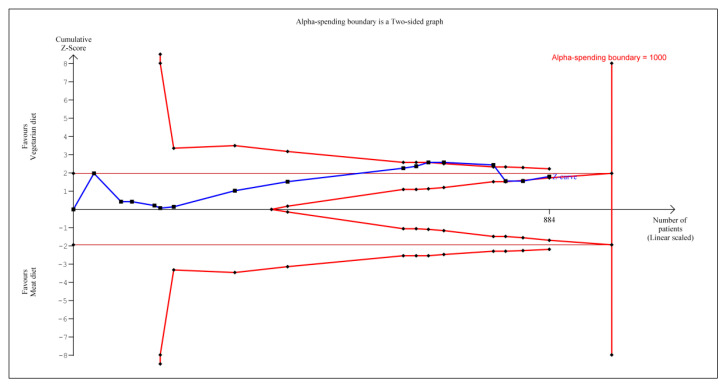
Trial sequential analysis on the effect of vegetarian diets vs. omnivorous diet on systolic blood pressure reduction.

According to TSA, the cumulative Z-curve (blue curve) shown in Figure 2 initially crossed the conventional boundary (Z-statistic above 1.96), based on the results of several trials [43,45,46,47,48,49,50,51,52,54,55,56]. However, it did not cross the conventional boundary when cumulating the results from the new trials [44,53,57] and demonstrated that vegetarian diets did not significantly reduce systolic blood pressure. However, the number of patients included in trial sequential analysis did not exceed the required information size (that is, 1000 patients), indicating that the cumulative evidence for vegetarian diets not reducing systolic blood pressure remains inconclusive based on these 884 participants.

### 3.4. Effect of Vegetarian Diets on Diastolic Blood Pressure Lowering and Its Subgroup Analysis

The effect of vegetarian diets on diastolic blood pressure lowering and its subgroup analysis by diet subgroup, diabetes status, and country are shown in Table 2. A significant reduction in diastolic blood pressure was observed in those on vegetarian diets compared to those on a control diet (WMD = −1.687, 95% CI = −2.968, −0.407) among all participants, regardless of age group. A similar result was also obtained among adult participants (WMD = −1.654, 95% CI = −2.958, −0.351). In subgroup analysis, we observed that a vegan diet (WMD = −1.920, 95% CI = −3.180, −0.661) had a reduction in diastolic blood pressure, whereas a lacto-ovo-vegetarian diet brings no changes in terms of BP reduction at the end of the trial. (WMD = −0.000, 95% CI = −0.000, 0.000). Surprisingly, the reduction in diastolic blood pressure reduction was greater among participants with diabetes (WMD = −1.838, 95% CI = −3.304, −0.373) as compared to those without diabetes (WMD = −1.242, 95% CI = −2.551, 0.066).

**Figure 3:**
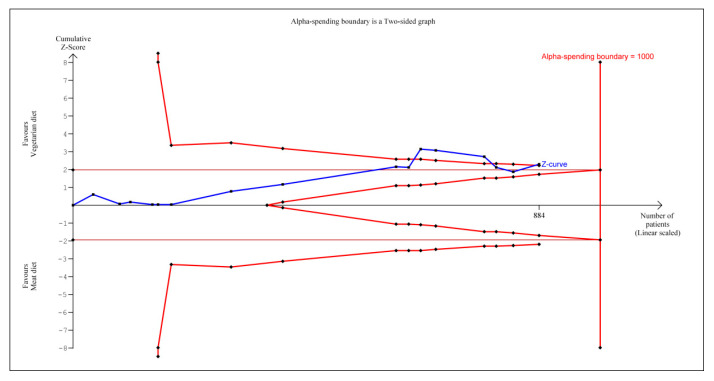
Trial sequential analysis on the effect of vegetarian diets vs. omnivorous diet on diastolic blood pressure reduction.

According to TSA, the cumulative Z-curve (blue curve) shown in Figure 3 initially crossed the conventional boundary (Z-statistic above 1.96) based on the results of several trials [43,45,46,47,48,49,50,51,52,54,55,56]. However, once again it did not cross the conventional boundary when cumulating the results from the new trials [44,53]. Later, one Z-curve crossed the conventional boundary again by cumulating the result from Wright et al. [57] and demonstrated that vegetarian diets are now associated with significant reduction in diastolic blood pressure. However, the number of patients included in trial sequential analysis did not exceed the required information size (that is, 1000 patients), indicating that the cumulative evidence for whether a vegetarian diet can reduce diastolic blood pressure remains inconclusive based on these 884 participants.

### 3.5. Risk of Bias within Studies

Risk of bias (Figures S11 and S12) was assessed under various categories such as bias arising from the randomization process, bias due to deviations from intended interventions, bias due to missing outcome data, bias in measurement of the outcome, and bias in selection of the reported result. All included studies were randomized controlled trials, but some studies gave cause for some concern of bias arising from the randomization process due to no information on how the randomization of sequence was conducted, how allocation sequences were concealed, and what baseline differences there were between intervention groups, suggesting a possible problem [46,47]. Studies with no information [51,54] and in which allocation sequences were not concealed [44] caused some concern, even though the random component was used in the sequence generation process. All included studies [43,44,45,46,47,48,49,50,51,52,53,54,55,56,57] had unclear risk of bias to deviation from intended intervention due to participants becoming aware of intervention, and there was no information on whether carers and people delivering the interventions were aware of participants’ assigned intervention during the trial. In addition, the diets taken by participants in both arms were either prepared by themselves with a guided cooking manual [43,44,45,46,48,49,50,53,54,55,56,57], or recipes throughout the trials and diets taken by participants were prepared at the investigation sites in a total of three trials [47,51,52], thus these studies cause some concern for bias due to deviation from intended intervention.

In the aspect of bias in outcomes measurement, the majority of the trials are at low risk of bias, except for one trial [43], which has allocation sequences that were not concealed. In the section for selection of the reported results, all studies had low risk of bias because the methods of measuring the outcome were appropriate and measurement of the outcome was the same between the intervention and control groups. For appraisal of bias in the selection of the reported results, all studies displayed low risk of bias because the data that produced this result were analyzed in accordance with a prespecified analysis plan, and there were no multiple outcome measurements for blood pressure within the outcome domain.

### 3.6. GRADE Assessment

A summary of the GRADE assessments of the overall certainty of the evidence for the effect of vegetarian diets on blood pressure lowering is demonstrated in Table S3. The evidence was graded as very low for systolic and diastolic blood pressure owing to a downgrade for risk of bias and inconsistencies. A downgrade for risk of bias might be because none of the included studies was ruled out for low risk of bias in those five bias assessments domains. There was evidence of very serious inconsistencies according to the I^2^ value, which indicated there are considerable proportions of variations due to interstudy differences.

## 4. Discussion

From the 15 RCT that are included in our study, we noticed that the composition of interventional diet in each study shows slight differences. For the Bernard study in 2009, the vegan diet with vitamin B12 supplementation consisted of vegetables, fruit, grains, and legumes, and was high in fiber and low in fat while the quantity, energy, and carbohydrate intake were unrestricted [43]. The study from the same author in 2017 introduced the same diet composition too [44]. Bloomer et al., 2015 used the “traditional” vegan-based Daniel fast diet that eliminates all processed foods and animal products with no restrictions on the portion sizes of food [45]. Both the GEICO studies (Ferdowsian et al. and Mishra et al.) of South Korea [46,50], Macknin et al. [49] and Nicholson et al. [51] followed a similar low-fat vegan diet as practiced in Bernard’s study. The study from Hunt et al. was used a lacto-ovo-vegetarian diet, which consisted of legumes, whole-grain bread and cereal products, and greater amounts of fruit and vegetables, and it had 25% less protein, 12% less fat, 16% more carbohydrate, 21% more ascorbic acid, slightly less saturated fat and <100 mg/d less cholesterol than the nonvegetarian diet [47]. Prescott et al.’s [52] interventional diet followed the diet of Seventh Day Adventist vegetarians studied in Rouse et al. [58]. In Ramal et al.’s study [53], the plant-based diet was based on the 30-Day Diabetes Miracle Cookbook [59] and was modified to suit the ethnic groups. Both Australian trials, namely Rouse et al. [54] and Sciarrone et al. [55] required those with lacto-ovo-vegetarian diet to maintain the intake of salt, eggs, and milk products with their routine diet patterns, with the menu and guidelines modified from the NUTRIVIEW program version 1.1. For Toobert et al.’s study [56], subjects were required to adhere to Reversal Diet guidelines [60], which contain no animal products other than egg whites, and non-fat yogurt with no added oils or other concentrated fats. Lastly the New Zealand study [57] on interventional diet was based on the whole food plant-based (WFPB) diet by the McDougall Program, which is also low in fat [61].

This meta-analysis revealed that a vegetarian dietary pattern significantly reduced systolic and diastolic blood pressure by WMD −2.655 and WMD −1.687, respectively. In general, this study yielded a similar finding to a previous meta-analysis of seven randomized controlled trials and thirty-two observational studies, which showed that the WMD in consumption of vegetarian diets versus omnivorous diet was systolic blood pressure of −4.8 mmHg in controlled trials and −6.9 mmHg in observational studies [24], while for diastolic blood pressure, the differences were −2.2 mmHg in controlled trials and −4.7 mmHg in observational studies [24]. The similar pattern in reduction of blood pressure in participants that received vegetarian diets as observed in the current study and previous study are definitely important to people with underlying diseases whom are currently not on a regular vegetarian diet, because the previous study indicated that by lowering 1 mmHg of systolic blood pressure it could reduce the incidence of coronary heart disease by 13.5 events, stroke by 12.1 events, and heart failure by 20.3 events per 100,000 person-years [62]. Moreover when systolic blood pressure was lowered by 2 mmHg, it substantially reduced the risk of cardiovascular diseases (27 events for coronary heart disease; 24.2 events for stroke; and 40.6 events for heart failure per 100,000 person-years) [62]. One of the possible mechanisms associated with the reduction in blood pressure seen with a vegetarian diet could be overwhelmingly due to its lowering effect of total cholesterol, low-density lipoprotein cholesterol, high-density lipoprotein cholesterol, and non-high-density lipoprotein cholesterol [63]. This is because the vegetarian diet is high in fiber, and omega-6 polyunsaturated fatty acids but low in cholesterol, total fat and saturated fatty acids as compared with omnivorous diet [64]. With a lower cholesterol level, this further helps to regulate the blood pressure [65]. The vegetarian diet is also rich in phytochemicals and antioxidants [64]. Previous studies have demonstrated that diets rich in fruits, vegetables, and whole grains, which lead to an increase in blood antioxidant capacity, can reduce systolic and diastolic blood pressure in both hypertensive and normotensive patients [66,67].

Among the various vegetarian diets, there was a difference result between the vegan and lacto-ovo-vegetarian diets. Greater reduction of BP was observed in the vegan diet. There was a greater BP reduction for the vegan diet (WMD = −3.118, 95% CI = −4.540, −1.696) as well as greater reduction in systolic blood pressure in the lacto-ovo-vegetarian diet (WMD = −1.752, 95% CI = −5.382, 1.878) compared with the omnivorous diet. This can be explained by the fact that regular consumption of meat, especially red meat, also increases the risk of falling ill because the processed derivatives in meat products are an associated factor for hypertension [68,69,70]. Besides, vegetarians have a significantly lower ischemic heart disease mortality (29%) [71]. These results are in accordance with a large cohort study conducted in America and Canada which indicated that the odds ratio for developing hypertension was lesser for vegetarian diets compared with an omnivorous diet: 0.37 (95% CI 0.19, 0.74) for vegans, 0.57 (95% CI 0.36, 0.92) for lacto-ovo-vegetarians and 0.92 (95% CI 0.50, 1.70) for partial vegetarians [72].

This finding suggested that dietary patterns based solely on plant sources are more effective in reducing blood pressure than other types of vegetarian diet which still include animal products or byproducts, such as eggs and dairy products, for example, in the lacto-ovo-vegetarian diet. This result is supported by previous studies in which high consumption of fruit and vegetables are associated with reduction of blood pressure when compared with those with a higher intake of dairy products [73,74,75]. One of the possible explanations for such an observation could be the higher content of saturated fatty acids in dairy products that affect the blood lipid profile by increasing low-density lipoprotein cholesterol and promoting atherosclerosis that might have contributed to higher blood pressure [76]. However, it should be noted that it is difficult to compare the effect of different types of the vegetarian diet in this study due to the different sample characteristics of each of these studies, therefore we should interpret the beneficial effect of vegetarian diet on blood pressure with great caution.

Even though vegetarian diets significantly improved both systolic and diastolic blood pressure, higher systolic blood pressure reduction was observed among subjects without diabetes (WMD = −4.083, 95% CI = −7.684, −0.482) compared to those with diabetes (WMD = −1.625, 95% CI = −3.106, −0.144). An opposite trend was observed with diastolic blood pressure in which higher diastolic blood pressure reduction was observed among subjects with diabetes (WMD = −1.838, 95% CI = −3.304, −0.373) compared to those without diabetes (WMD = −1.242, 95% CI = −2.551, 0.066). It could be because the mean age of the diabetic group (54.9 years old) was much older than the nondiabetic group (40.9 years old), and thus the diastolic BP reduction was not much observed among the younger nondiabetic group.

Therefore, vegetarian diets have the potential to be recommended to those with underlying disease as well as the general population, in the hope that, through this nonpharmacological diet method, uncontrolled hypertension could be reduced, which would result in a meaningful decrease in the risk of developing cardiovascular diseases as well as other morbidities and mortality and lessen the excessive demand on healthcare.

We also performed the meta-analysis with trial sequential analysis to estimate the effect vegetarian diet had on BP reduction. To provide insight into the power of sample size to conclude the findings, the number of patients included in trial sequential analysis did not exceed the required information size (that is, 1000 patients). Similar findings are applied to both systolic and diastolic blood pressure as the number of patients included in trial sequential analysis also similarly did not exceed the required information size (that is, 1000 patients). This indicated the cumulative evidence on whether vegetarian diets can reduce systolic and diastolic blood pressure remains inconclusive based on these 884 participants.

### Limitations and Future Research Recommendation

The current meta-analysis has several limitations. Firstly, there was high heterogeneity among the controlled trials included in the analysis. The heterogeneities across studies could be due to participant characteristics, such as some of the trials involved participants with lifestyle restriction practices including limiting alcohol, salt, fatty food, caffeine intake, and/or cigarette smoking while others did not. These are confounding factors to blood pressure control, thus, it may affect the findings of the study.

Secondly, this study only includes dietary patterns, instead of isolated nutrients. There was a lack of information on the nutrition value of various diet regiments consumed by participants in those trials in both the interventional and control arms. Hence, it may cause a plausible application of the results to both general and clinical populations. Only with the availability of the nutritional value of these diets, could we then give recommendations regarding the specific quantities of these foods that should be consumed for the best positive effect. It is important to know energy intake could be an important factor influencing body weight and blood pressure reduction; however, there was also a lack of information on comparisons of changes from baseline to final values for energy intake and body weight between vegetarian diets compared with control diets. Therefore, we could not entangle energy intake on blood pressure lowering in the current study.

Thirdly, the included studies were mainly conducted in the USA and other Western countries (Australia and New Zealand). Hence, it cannot be generalized to other populations. Additionally, heavy metals can be found both in animal-derived food or plant-based food. However, sometimes it may be higher in the latter one as they can be easily contaminated with heavy metals such as arsenic, cadmium and lead, from the water, air, and soil as they grow [77,78,79,80]. These heavy metals have been found to inactivate catechol-O-methyltransferase (COMT), which increases serum and urinary epinephrine, norepinephrine, and dopamine [81]. This resulted in hormone imbalances, vasoconstriction, and affected renal tubular function, which eventually leads to hypertension [82].

Fourthly, as mentioned previously, the sample size gathered from all clinical trials done in the past has not yet reached the required sample size for drawing a conclusive statement. Therefore, we suggest that, in future, larger randomized clinical trials aimed at examining the effect of the vegetarian diet on blood pressure be conducted, paying attention to publication bias. Furthermore, the number of studies is small and any derived conclusions should be made with caution.

## 5. Conclusions

Notwithstanding these limitations, this study suggests that, evidence from clinical trials has shown that vegetarian diets, especially vegan diets, reduce blood pressure when compared with omnivorous diets, suggesting that they may be crucial in the primary prevention and overall management of hypertension.

## Figures and Tables

**Table 1:** Characteristics of trial.

First Author (Vegetarian vs. Control)	Year	Area	Age, Mean ± SD (Range)		Gender, *n* (%)		Medication (%)		Baseline BMI, Mean ± SD (Range)		Energy Intake Difference ^†^	Weight Difference ^†^	Diabetes	Vegetarian/Vegan	Control Group	Trial Duration
			Vegan	Conventional Diet	Vegan	Conventional Diet	Vegan	Conventional Diet	Vegan	Conventional Diet						
Barnard et al., 2009 (Low-fat vegan diet vs. conventional diabetes diet)	2013	USA	56.7 ± 9.8 (35–82)	54.6 ± 10.2 (27–80)	Male, 22 (45); Female, 27 (55)	Male, 17 (34); Female, 33 (66)	DM (78); HPT (76); LIPID (54)	DM (69); HPT (63); LIPID (55)	N/A	N/A	0.90	0.25	With Diabetes	Vegan	meat diet	74 weeks
Barnard et al., 2017 (Low-fat vegan diet vs. portion-controlled eating plan)	2017	USA	61 (41–79)	61 (30–75)	Male, 8 (38); Female, 13 (62)	Male, 13 (54); Female, 11 (46)	N/A	N/A	34.9 ± 1.5	33.0 ± 1.3	0.46	0.10	With Diabetes	Vegan	Meat diet	20 weeks
Bloomer et al., 2015 (Traditional Daniel fast vs. Modified Daniel fast)	2015	USA	33 ± 2 years (18–67 years)		Male, 6 (17.1); Female, 29 (82.9)		N/A	N/A	26.2 ± 1.3 (19–45)	25.6 ± 1.4 (19–45)	0.04	>0.05	Without Diabetes	Vegan	Meat diet	3 weeks
Ferdowsian et al., 2010 (low-fat vegan diet vs. control diet)	2010	USA	44.4 (21–65)		Male, 20 (17.7); Female, 93 (82.3)		N/A	N/A	N/A	N/A	0.017	<0.0001	With Diabetes	Vegan	Meat diet	22 weeks
Hunt et al., 1998 (Lacto-ovo-vegetarian vs. non-vegetarian diet)	1998	USA	33 ± 7 (20–42)		Female, 21 women (100)		No, with the exception that 9 used hormonal contraceptives		23.5 ± 2.8 (range: 19.0–29.0)		N/A	N/A	Without Diabetes	Lacto-ovo-vegetarian	Meat diet	8 weeks
Lee et al., 2016 (Brown rice vegan diet vs. conventional diabetic diet)	2016	South Korea	57.5 ± 7.7 (32–70)	58.3 ± 7.0 (40–69)	Male, 6 (13.0); Female, 40 (87.0)	Male, 12 (25.5); Female, 35 (74.5)	DM (73.9); HPT (39.1); LIPID (50)	DM (76.6); HPT (46.8); LIPID (55.3)	23.9 ± 3.4	23.1 ± 2.4	0.042	N/A	With Diabetes	Vegan	Meat diet	12 weeks
Macknin et al., 2015 (Plant-based low fat diet vs. American Heart Association diets)	2015	USA	15.0 (9.0–18.0)	15.0 (9.0–18.0)	Male, 5 (36); Female, 9 (64)	Male, 5 (36); Female, 9 (64)	N/A	N/A	Overweight, 4 (29%); Obese, 10 (71%)	Overweight, 2(14%); Obese, 12 (86%)	N/A	N/A	Without Diabetes	Vegan	Meat diet	4 weeks
Mishra et al., 2013 (low-fat vegan diet vs. control diet)	2013	USA	44.3 ± 15.3	46.1 ± 13.6	Male, 32 (23); Female, 110 (77)	Male, 18 (12); Female, 132 (88)	N/A	N/A	34.7 ± 0.6	35.3 ± 0.7	0.09	<0.001	With Diabetes	Vegan	Meat diet	18 weeks
Nicholson et al., 1999 (low-fat Vegan diet vs. control diet)	1999	USA	Mean 54.3		Male, (54.5); Female (45.5)		81.80		N/A		N/A	N/A	With Diabetes	Vegan	Meat diet	12 weeks
Prescott et al., 1987 (Lacto-ovo-vegetarian vs. non-vegetarian diet)	1987	Australia	36.4 ± 2.4	34.0 ± 2.1	Male, 9; Female, 16	Male, 11; Female, 14	N/A	N/A	25.3 ± 0.9	25.5 ± 1.0	N/A	N/A	Without Diabetes	Lacto-ovo-vegetarian	Meat diet	12 weeks
Ramal et al., 2017 (High-fiber low-fat plant based diet vs. control diet)	2017	USA	53.35 ± 6.74	52.93 ± 13.11	Male, 4 (23.5); Female, 13 (76.5)	Male, 3 (20.0); Female, 12 (80.0)	15 (88.2)	13 (86.7)	31.81 ± 1.01	30.84 ± 1.08	N/A	N/A	With Diabetes	Vegan	Meat diet	24 weeks
Rouse et al., 1986 (Lacto-ovo-vegetarian vs. non-vegetarian diet)	1986	Australia	Mean 40.1		Male (50); Female (50)		no		23.7		N/A	N/A	Without Diabetes	Lacto-ovo-vegetarian	Meat diet	14 weeks
Sciarrone et al., 1993 (Lacto-ovo-vegetarian vs. non-vegetarian diet)	1993	Australia	Mean 41		Male (100)		no		25.3		N/A	N/A	Without Diabetes	Lacto-ovo-vegetarian	Meat diet	6 weeks
Toobert et al., 2000 (Prime time diet vs. Usual care diet)	2000	USA	64 ± 10	63 ± 11	Female, 25 (100)		ERT (35.7); HPT (71.4); LIPID (28.6)	ERT (45.5); HPT (81.8); LIPID (45.5)	32 ± 4.2	32 ± 5.5	N/A	N/A	Without Diabetes	Lacto-ovo-vegetarian	Meat diet	24 months
Wright et al., 2017 (Low-fat plant-based diet vs. control diet)	2017	New Zealand	56 ± 9.9	56 ± 9.5	Male, 11 (33); Female, 22 (67)	Male, 15 (47); Female, 17 (53)	N/A	N/A	34.5 ± 1.6	34.2 ± 2.3	N/A	N/A	With Diabetes	Vegan	Meat diet	48 weeks

Data are presented in either mean ± standard deviation (SD), range or *n* (%). Additional information on characteristics of the trials is presented in Table S2. ^†^ Refers to *p* value for *t* tests for between-group (vegetarian diets compared with control diets) comparisons of changes from baseline to final values. vs. = versus; N/A = Not available.

**Table 2:** Weighted mean difference and 95% confidence interval of blood pressure by subgroup analysis.

Variables		N	Weighted Mean Difference	95% CI	I^2^	*p*-Value	Forest Plot
**Systolic blood pressure**							
Overall systolic blood pressure (inclusive of children)		16	−2.655	(−3.758, −1.553)	98.32	<0.001	Figure S1
Overall systolic blood pressure (exclusive of children)		15	−2.509	(−3.630, −1.388)	98.42	<0.001	Figure S2
Diet subgroup	Vegan diet	11	−3.118	(−4.540, −1.696)	96.99	<0.001	Figure S3
	Lacto-ovo-vegetarian diet	5	−1.752	(−5.382, 1.878)	72.69	0.005	
Diabetes subgroup	Participants with diabetes	8	−1.625	(−3.106, −0.144)	96.84	<0.001	Figure S4
	Participants without diabetes	8	−4.083	(−7.684, −0.482)	96.90	<0.001	
Country	USA	11	−2.803	(−4.037, −1.569)	98.85	<0.001	Figure S5
	Australia	3	−2.075	(−9.859, 5.709)	83.24	0.003	
	New Zealand	1	−4.000	(−6.352, −1.648)	N.A	N.A	
	South Korea	1	1.000	(−3.306, 5.306)	N.A	N.A	
**Diastolic blood pressure**							
Overall diastolic blood pressure (inclusive of children)		16	−1.687	(−2.968, −0.407)	99.35	<0.001	Figure S6
Overall diastolic blood pressure (exclusive of children)		15	−1.654	(−2.958, −0.351)	99.39	<0.001	Figure S7
Diet subgroup	Vegan diet	11	−1.920	(−3.180, −0.661)	97.80	<0.001	Figure S8
	Lacto-ovo-vegetarian diet	5	−0.000	(0.000, 0.000)	0.0	0.432	
Diabetes subgroup	Participants with diabetes	8	−1.838	(−3.304, −0.373)	98.46	<0.001	Figure S9
	Participants without diabetes	8	−1.242	(−2.551, 0.066)	57.48	0.021	
Country	USA	11	−2.179	(−3.678, −0.680)	99.57	<0.001	Figure S10
	Australia	3	−0.302	(−2.912, 2.308)	0.0	0.468	
	New Zealand	1	−1.000	(−2.176, 0.176)	N.A	N.A	
	South Korea	1	1.100	(−1.501, 3.701)	N.A	N.A	

## Supplementary Materials

The following are available online at https://www.mdpi.com/2072-6643/12/6/1604/s1, Figure S1. Forest plot for overall systolic blood pressure (inclusive of children). Figure S2. Forest plot for overall systolic blood pressure (exclusive of children). Figure S3. Forest plot for overall systolic blood pressure (diet subgroup). Figure S4. Forest plot for overall systolic blood pressure (diabetes subgroup). Figure S5. Forest plot for overall systolic blood pressure (country subgroup). Figure S6. Forest plot for overall diastolic blood pressure (inclusive of children). Figure S7. Forest plot for overall diastolic blood pressure (exclusive of children). Figure S8. Forest plot for overall diastolic blood pressure (diet subgroup). Figure S9. Forest plot for overall diastolic blood pressure (diabetes subgroup). Figure S10. Forest plot for overall diastolic blood pressure (country subgroup). Figure S11. Risk of bias graph shows review authors’ judgements about each risk of bias items presented as percentages across all included studies. Figure S12. Risk of bias summary shows review authors’ judgements about each risk of bias items for each included studies. Table S1. Search terms used for final search on 6 January 2020. Table S2. Additional information on characteristics of trials. Table S3. GRADE quality assessment for the study findings was summarised as followed.
