# Supplementary material for: Effects of Vegetarian Diets on Blood Pressure Lowering: A Systematic Review with Meta-Analysis and Trial Sequential Analysis
Source: Nutrients. 2020 May 29;12(6):1604. doi: 10.3390/nu12061604 (PMC7352826; doi:10.3390/nu12061604)
Supplement: Supplementary file 1 [file nutrients-12-01604-s001.pdf]

Table S1. Search terms used for final search on 6 January 2020.

Searches	Search terms	Medline	PubMed	Cochrane Central Register of controlled trials
#1	exp diet OR vegetarian OR vegan OR lacto-ovo vegetarian OR lacto vegetarian OR plant based diet OR vegetarianism OR ornish diet OR CHIP diet OR pesco vegetarian OR semi vegetarian	273649	21841	17313
#2	exp hypertension	250399	11251	16856
#3	exp blood pressure	286562	18281	26961
#4	exp randomized controlled trials as Topic/	132629	740	7732
#5	exp clinical trial/	848231	14122	163
#6	random$.ab.	1067633	262582	803155
#7	1 AND 2	8109	795	728
#8	1 AND 3	8056	936	1363
#9	4 OR 5 OR 6	1593142	276410	805284
#10	7 OR 8	13058	1165	1686
#11	9 AND 10	2818	75	1255
#12	limit 11 to (english language and humans)	2593	72	1243

Table S2. Additional information on characteristics of trials.

First Author (vetarian vs control)	Year	Area	Participants	alcohol permitted	Salt permitted	Fatty food permitted	Caffeine permitted	Cigarette permitted	Intention to treat (veg)	Per protocol (veg)	completion %	Intention to treat (control)	Per protocol (control)	completion %
Barnard et al., 2009 (Low-fat vegan diet vs conventional diabetes diet)	2013	USA	Free-living individuals with type 2 diabetes	Yes	Yes	No	Unsure	Unsure	49	40	81.6	50	43	86
Barnard et al., 2017 (Low-fat vegan diet vs portion-controlled eating plan)	2017	USA	Individuals with T2DM	Yes	Yes	Yes	Unsure	Unsure	21	19	90.5	24	21	87.5
Bloomer et al., 2015 (Traditional Daniel fast vs Modified Daniel fast)	2015	USA	Free-living individuals (6 men: 30 women), average age of 33 ± 2 years and a range of 18–67 years	No	No	No	No	No	12	12	100	12	12	100
Ferdowsian et al., 2010 (low-fat vegan diet vs control diet)	2010	USA	Individuals with BMI ≥ 25 and or diabetes, aged 21-65 years	No	Unsure	No	Unsure	unsure	68	65	95.9	45	44	97.8
Hunt et al., 1998 (Lacto-ovo-vegetarian vs non-vegetarian diet)	1998	USA	21 Healthy women aged 33±7 years (ranged 20-42 years)	Unsure	Yes	Yes	No	unsure	21	21	100	21	21	100
Lee et al., 2016 (Brown rice vegan diet vs conventional diabetic diet)	2016	South Korea	Individual with T2DM (age ranged 32-70)	Unsure	Yes	Yes	Unsure	Unsure	53	46	86.8	53	47	88.7
Macknin et al., 2015 (Plant-based low fat diet vs American Heart Association diets)	2015	USA	Children (aged 9-18 years)	Unsure	Unsure	Unsure	Unsure	Unsure	16	14	87.5	14	14	100
Mishra et al., 2013 (low-fat vegan diet vs control diet)	2013	USA	Individuals with BMI ≥ 25 and or diabetes	No	Unsure	No	Unsure	unsure	142	94	66.2	149	117	78.5

Nicholson et al., 1999 (low-fat Vegan diet vs control diet)	1999	USA	Individuals with diabetes	No	Unsure	Yes	Unsure	No	7	7	100	6	4	66.7
Prescott et al., 1987 (Lacto-ovo-vegetarian vs non-vegetarian diet)	1987	Australia	Male and female omnivores, aged 18-60 years, not being treated for hypertension were recruited from hospital staff	Yes	Yes	Unsure	Yes	Yes	32	25	78.1	32	25	78.1
Ramal et al., 2017 (High-fiber low-fat plant based diet vs control diet)	2017	USA	Free-living adults Latino with T2DM	Yes	Unsure	Yes	Yes	Yes	19	17	89.5	19	15	78.9
Rouse et al., 1986 (Lacto-ovo-vegetarian vs non-vegetarian diet)	1986	Australia	Healthy free-living omnivore	Yes	Yes	Yes	Unsure	Unsure	20	20	100	20	19	95
Sciarrone et al., 1993 (Lacto-ovo-vegetarian vs non-vegetarian diet)	1993	Australia	Normotensive men hospital employees (Aged 30-59 years)	Yes	Yes	Unsure	No	No	10	10	100	10	10	100
Toobert et al., 2000 (Prime time diet vs Usual care diet)	2000	USA	postmenopansal female and having documented CHD defined as atherosclerosis, myocardial infarction (MI), percutaneous transluminal coronaryangioplasty, life-threatening illnesses, infarction during the preceding 6 weeks,and/or coronary bypass graft surgery.	Unsure	Unsure	Unsure	Unsure	Unsure	14	14	100	14	11	78.6
Wright et al., 2017 (Low-fat plant-based diet vs control diet)	2017	New Zealand	Diagnosed with obesity or overweight and at least one of type 2 diabetes, ischaemic heart disease, hypertension or hypercholesterolaemia (aged 35-70 years)	No	Yes	No	Yes	No	33	25	75.8	32	24	75

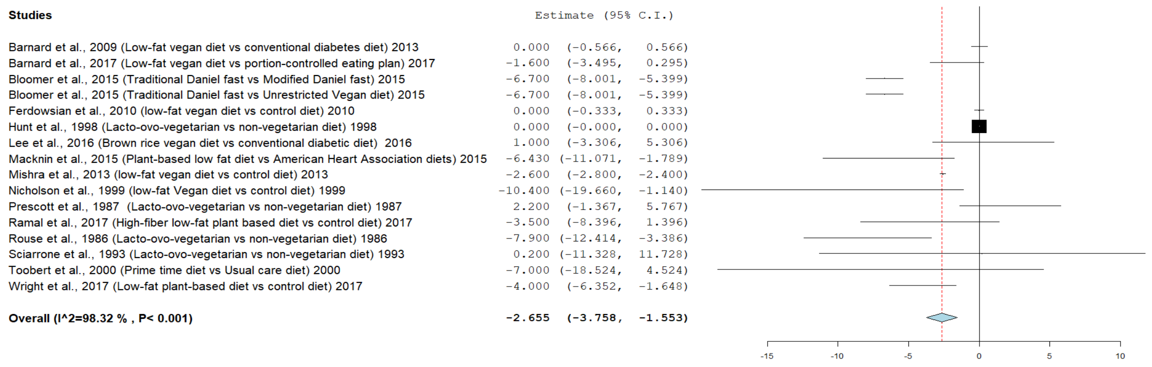

Figure S1. Forest plot for overall systolic blood pressure (inclusive of children).

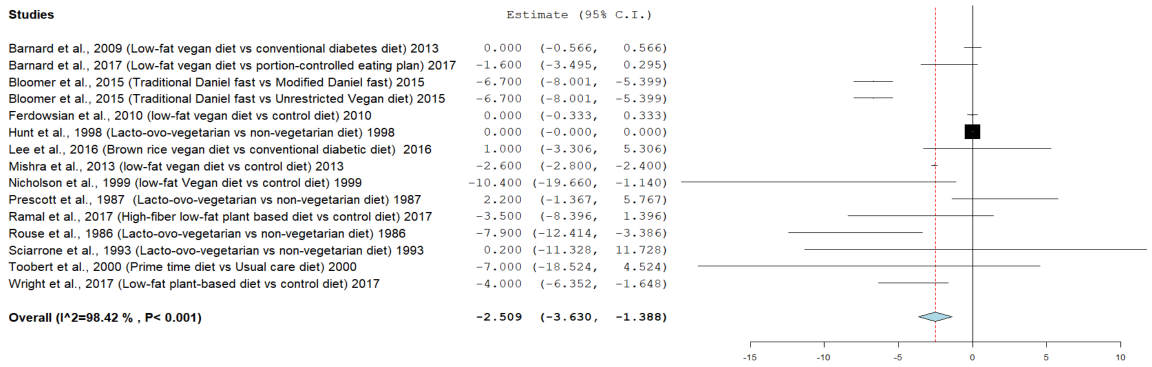

Figure S2. Forest plot for overall systolic blood pressure (exclusive of children).

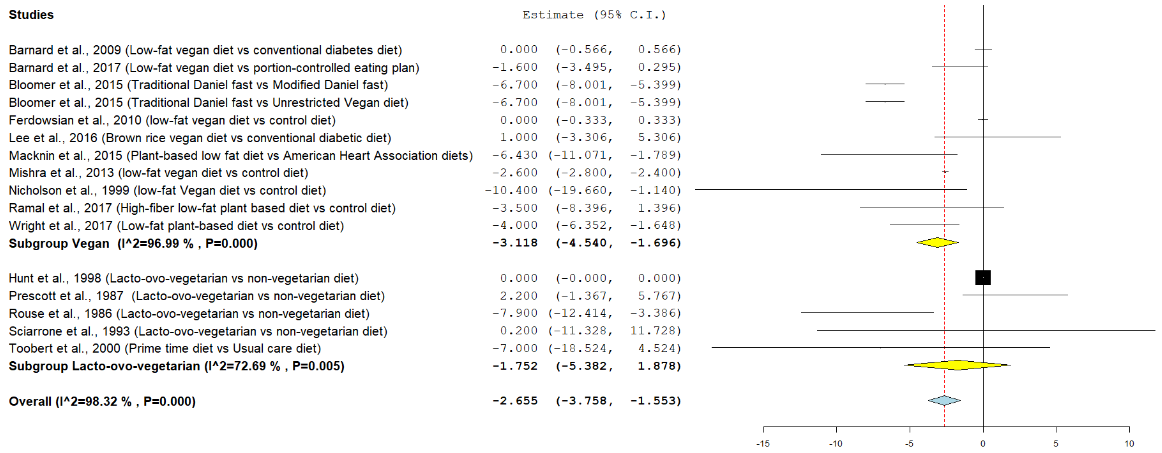

Figure S3. Forest plot for overall systolic blood pressure (diet subgroup).

Studies	Estimate (95% C.I.)		
Barnard et al., 2009 (Low-fat vegan diet vs conventional diabetes diet)	0.000	(-0.566,	0.566)
Barnard et al., 2017 (Low-fat vegan diet vs portion-controlled eating plan)	-1.600	(-3.495,	0.295)
Ferdowsian et al., 2010 (low-fat vegan diet vs control diet)	0.000	(-0.333,	0.333)
Lee et al., 2016 (Brown rice vegan diet vs conventional diabetic diet)	1.000	(-3.306,	5.306)
Mishra et al., 2013 (low-fat vegan diet vs control diet)	-2.600	(-2.800,	-2.400)
Nicholson et al., 1999 (low-fat Vegan diet vs control diet)	-10.400	(-19.660,	-1.140)
Ramal et al., 2017 (High-fiber low-fat plant based diet vs control diet)	-3.500	(-8.396,	1.396)
Wright et al., 2017 (Low-fat plant-based diet vs control diet)	-4.000	(-6.352,	-1.648)
**Subgroup With Diabetes (I^2=96.84 % , P=0.000)**	**-1.625**	**(-3.106,**	**-0.144)**
Bloomer et al., 2015 (Traditional Daniel fast vs Modified Daniel fast)	-6.700	(-8.001,	-5.399)
Bloomer et al., 2015 (Traditional Daniel fast vs Unrestricted Vegan diet)	-6.700	(-8.001,	-5.399)
Hunt et al., 1998 (Lacto-ovo-vegetarian vs non-vegetarian diet)	0.000	(-0.000,	0.000)
Macknin et al., 2015 (Plant-based low fat diet vs American Heart Association diets)	-6.430	(-11.071,	-1.789)
Prescott et al., 1987 (Lacto-ovo-vegetarian vs non-vegetarian diet)	2.200	(-1.367,	5.767)
Rouse et al., 1986 (Lacto-ovo-vegetarian vs non-vegetarian diet)	-7.900	(-12.414,	-3.386)
Sciarrone et al., 1993 (Lacto-ovo-vegetarian vs non-vegetarian diet)	0.200	(-11.328,	11.728)
Toobert et al., 2000 (Prime time diet vs Usual care diet)	-7.000	(-18.524,	4.524)
**Subgroup Without Diabetes (I^2=96.9 % , P=0.000)**	**-4.083**	**(-7.684,**	**-0.482)**
**Overall (I^2=98.32 % , P=0.000)**	**-2.655**	**(-3.758,**	**-1.553)**

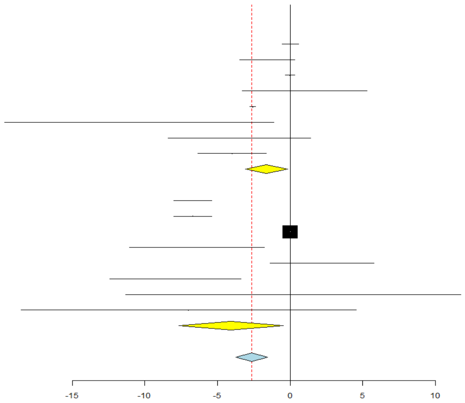

Figure S4. Forest plot for overall systolic blood pressure (diabetes subgroup).

Studies	Estimate (95% C.I.)		
Barnard et al., 2009 (Low-fat vegan diet vs conventional diabetes diet)	0.000	(-0.566,	0.566)
Barnard et al., 2017 (Low-fat vegan diet vs portion-controlled eating plan)	-1.600	(-3.495,	0.295)
Bloomer et al., 2015 (Traditional Daniel fast vs Modified Daniel fast)	-6.700	(-8.001,	-5.399)
Bloomer et al., 2015 (Traditional Daniel fast vs Unrestricted Vegan diet)	-6.700	(-8.001,	-5.399)
Ferdowsian et al., 2010 (low-fat vegan diet vs control diet)	0.000	(-0.333,	0.333)
Hunt et al., 1998 (Lacto-ovo-vegetarian vs non-vegetarian diet)	0.000	(-0.000,	0.000)
Macknin et al., 2015 (Plant-based low fat diet vs American Heart Association diets)	-6.430	(-11.071,	-1.789)
Mishra et al., 2013 (low-fat vegan diet vs control diet)	-2.600	(-2.800,	-2.400)
Nicholson et al., 1999 (low-fat Vegan diet vs control diet)	-10.400	(-19.660,	-1.140)
Ramal et al., 2017 (High-fiber low-fat plant based diet vs control diet)	-3.500	(-8.396,	1.396)
Toobert et al., 2000 (Prime time diet vs Usual care diet)	-7.000	(-18.524,	4.524)
**Subgroup USA (I^2=98.85 % , P=0.000)**	**-2.803**	**(-4.037,**	**-1.569)**
Lee et al., 2016 (Brown rice vegan diet vs conventional diabetic diet)	1.000	(-3.306,	5.306)
**Subgroup South Korea (I^2=NA , P=NA)**	**1.000**	**(-3.306,**	**5.306)**
Prescott et al., 1987 (Lacto-ovo-vegetarian vs non-vegetarian diet)	2.200	(-1.367,	5.767)
Rouse et al., 1986 (Lacto-ovo-vegetarian vs non-vegetarian diet)	-7.900	(-12.414,	-3.386)
Sciarrone et al., 1993 (Lacto-ovo-vegetarian vs non-vegetarian diet)	0.200	(-11.328,	11.728)
**Subgroup Australia (I^2=83.24 % , P=0.003)**	**-2.075**	**(-9.859,**	**5.709)**
Wright et al., 2017 (Low-fat plant-based diet vs control diet)	-4.000	(-6.352,	-1.648)
**Subgroup New Zealand (I^2=NA , P=NA)**	**-4.000**	**(-6.352,**	**-1.648)**
**Overall (I^2=98.32 % , P=0.000)**	**-2.655**	**(-3.758,**	**-1.553)**

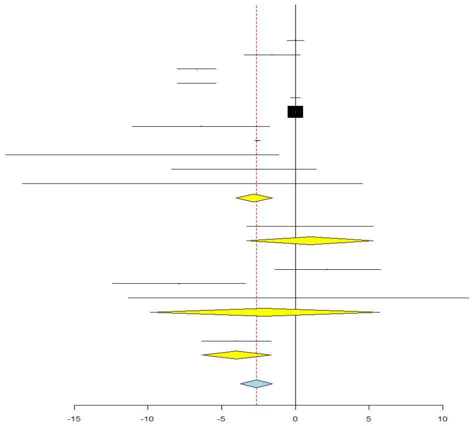

Figure S5. Forest plot for overall systolic blood pressure (country subgroup).

Studies	Estimate (95% C.I.)		
Barnard et al., 2009 (Low-fat vegan diet vs conventional diabetes diet) 2013	-3.900	(-4.268,	-3.532)
Barnard et al., 2017 (Low-fat vegan diet vs portion-controlled eating plan) 2017	-1.200	(-3.421,	1.021)
Bloomer et al., 2015 (Traditional Daniel fast vs Modified Daniel fast) 2015	-2.100	(-3.797,	-0.403)
Bloomer et al., 2015 (Traditional Daniel fast vs Unrestricted Vegan diet) 2015	-2.100	(-3.797,	-0.403)
Ferdowsian et al., 2010 (low-fat vegan diet vs control diet) 2010	-0.400	(-0.661,	-0.139)
Hunt et al., 1998 (Lacto-ovo-vegetarian vs non-vegetarian diet) 1998	0.000	(-0.000,	0.000)
Lee et al., 2016 (Brown rice vegan diet vs conventional diabetic diet) 2016	1.100	(-1.501,	3.701)
Macknin et al., 2015 (Plant-based low fat diet vs American Heart Association diets) 2015	-2.600	(-7.995,	2.795)
Mishra et al., 2013 (low-fat vegan diet vs control diet) 2013	-3.500	(-3.660,	-3.340)
Nicholson et al., 1999 (low-fat Vegan diet vs control diet) 1999	-5.800	(-11.060,	-0.540)
Prescott et al., 1987 (Lacto-ovo-vegetarian vs non-vegetarian diet) 1987	0.900	(-2.393,	4.193)
Ramal et al., 2017 (High-fiber low-fat plant based diet vs control diet) 2017	-1.200	(-4.195,	1.795)
Rouse et al., 1986 (Lacto-ovo-vegetarian vs non-vegetarian diet) 1986	-2.700	(-7.389,	1.989)
Sciarrone et al., 1993 (Lacto-ovo-vegetarian vs non-vegetarian diet) 1993	-0.500	(-10.975,	9.975)
Toobert et al., 2000 (Prime time diet vs Usual care diet) 2000	-6.000	(-13.857,	1.857)
Wright et al., 2017 (Low-fat plant-based diet vs control diet) 2017	-1.000	(-2.176,	0.176)
**Overall (I^2=99.35 % , P< 0.001)**	**-1.687**	**(-2.968,**	**-0.407)**

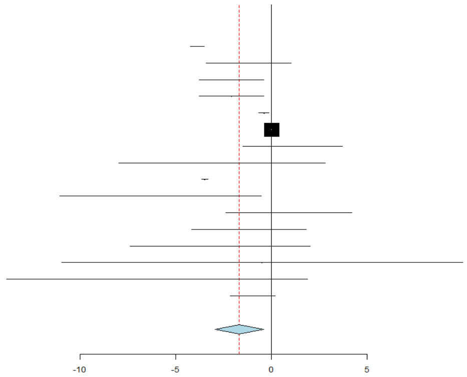

Figure S6. Forest plot for overall diastolic blood pressure (inclusive of children).

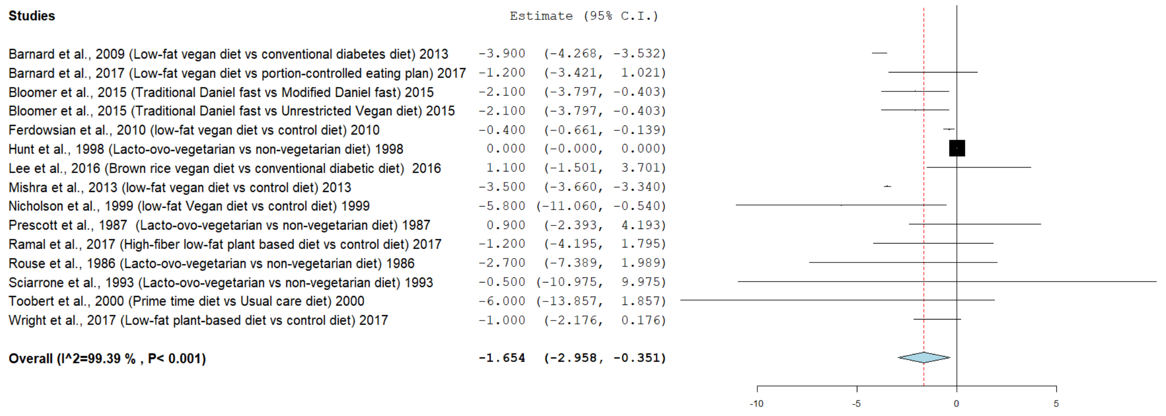

Figure S7. Forest plot for overall diastolic blood pressure (exclusive of children).

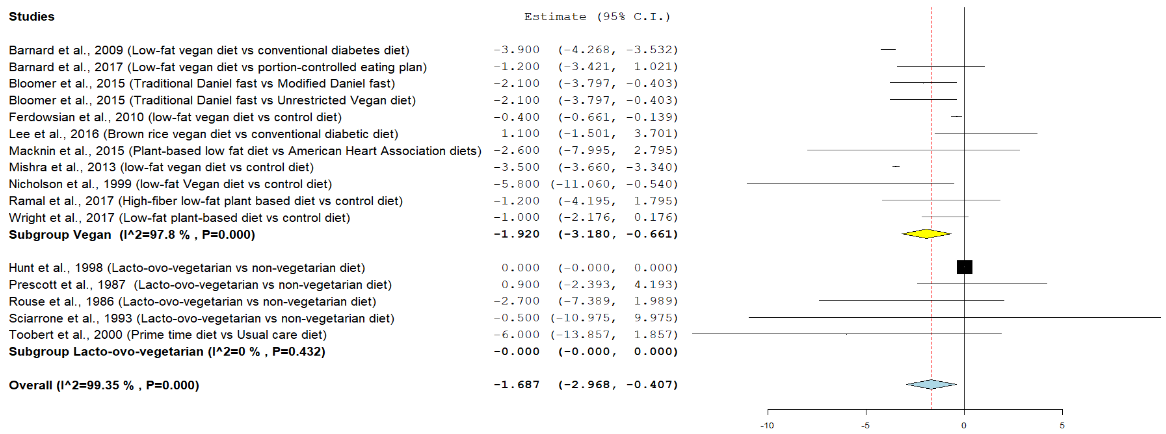

Figure S8. Forest plot for overall diastolic blood pressure (diet subgroup).

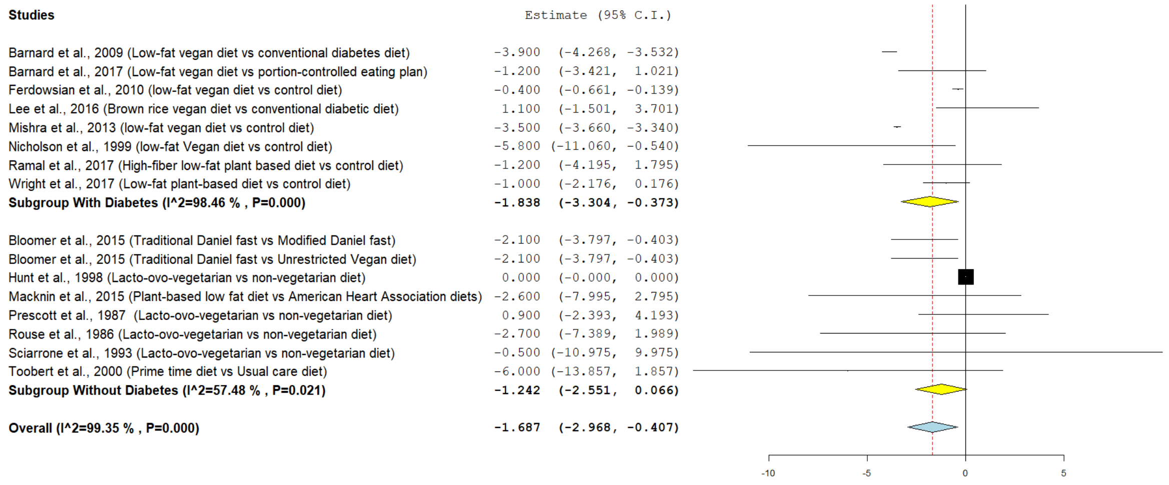

Figure S9. Forest plot for overall diastolic blood pressure (diabetes subgroup).

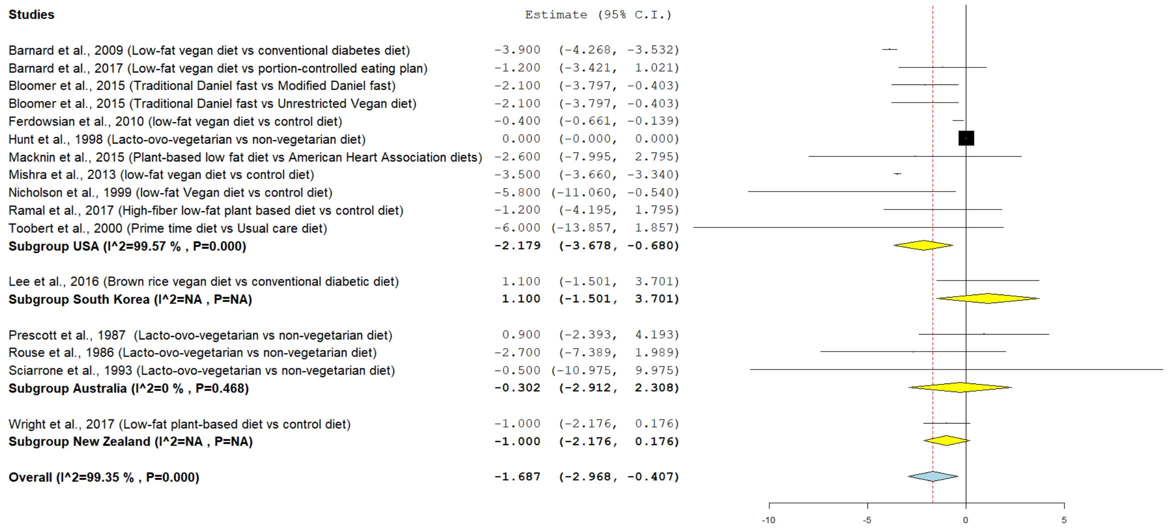

Figure S10. Forest plot for overall diastolic blood pressure (country subgroup).

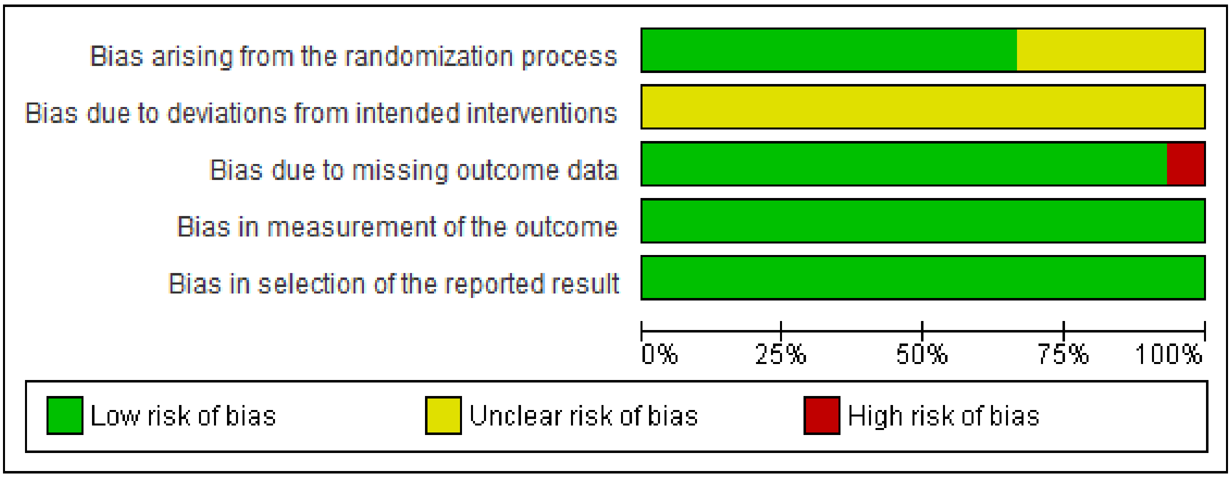

Figure S11. Risk of bias graph shows review authors' judgements about each risk of bias items presented as percentages across all included studies.

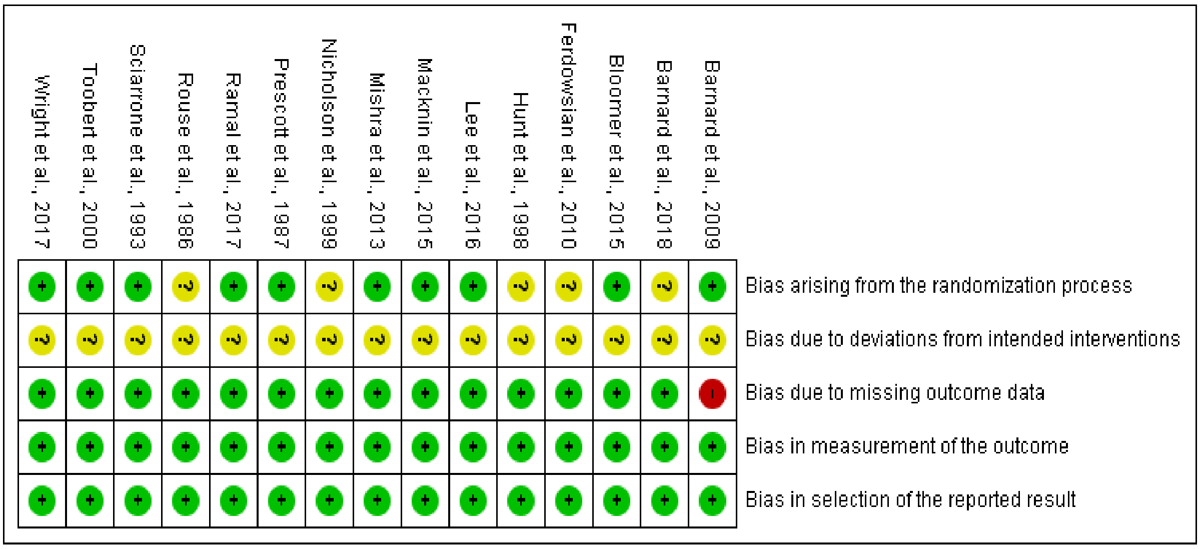

Figure S12. Risk of bias summary shows review authors' judgements about each risk of bias items for each included studies.

Table S3. GRADE quality assessment for the study findings was summarised as followed.

Certainty assessment							№ of patients		Effect			
**№ of studies**	**Study design**	**Risk of bias**	**Inconsistency**	**Indirectness**	**Imprecision**	**Other considerations**	**Vegetarian diets**	Ominorous diets	Relative (95% CI)	Absolute (95% CI)	Certainty	Importance
Systolic blood pressure lowering (follow up: range 3 weeks to 74 weeks; assessed with: mm Hg; Scale from: 0 to 200)												
16	randomised trials	serious	very serious	not serious	not serious	none	444	440	-	MD 2.655 lower (3.758 lower to 1.553 lower)	⨁◯◯◯ VERY LOW	
Diastolic blood pressure lowering (follow up: range 3 weeks to 74 weeks; assessed with: mm Hg; Scale from: 0 to 200)												
16	randomised trials	serious	very serious	not serious	not serious	none	444	440	-	MD 1.687 lower (2.968 lower to 0.407 lower)	⨁◯◯◯ VERY LOW	

CI: Confidence interval; **MD:** Mean difference
